# Elimination of onchocerciasis in Africa by 2025: the need for a broad perspective

**DOI:** 10.1186/s40249-019-0557-1

**Published:** 2019-07-15

**Authors:** Ed Cupp, Mauricio Sauerbrey, Vitaliano Cama, Mark Eberhard, Patrick J. Lammie, Thomas R. Unnasch

**Affiliations:** 1 Department of Entomology and Plant Pathology, Auburn University, Auburn, AL 36849 USA; 2 Onchocerciasis Elimination Program for the Americas, 14 Calle 3-51 Zona 10, Edificio Murano Center, Oficina, 1401 Guatemala City, Guatemala; 3 Centers for Disease Control and Prevention, 1600 Clifton Rd, MS D-65, Atlanta, GA 30329 USA; 4 Task Force for Global Health, 330 W. Ponce de Leon Ave, Decatur, GA 30030 USA; 5 Center for Global Health Infectious Diseases, University of South Florida College of Public Health, 3720 Spectrum Blvd, Suite 304, Tampa, FL 33612 USA

**Keywords:** Onchocerciasis, Ivermectin, Onchocerciasis in the Americas, Elimination, African Programme for onchocerciasis control, Onchocerciasis control program of West Africa

## Abstract

**Background:**

In response to the recent publication “Is onchocerciasis elimination in Africa feasible by 2025: a perspective based on lessons learnt from the African control programmes” by Dadzie et al., it is important to clarify and highlight the positive and unequivocal research and operational contributions from the American experience towards the worldwide elimination of human onchocerciasis (river blindness).

**Main text:**

The strategies of twice or more rounds of mass drug administration (MDA) of ivermectin per year, as well as the use of OV-16 serology have allowed four American countries to be verified by World Health Organization to have eliminated transmission of *Onchocerca volvulus*, the etiological agent. These advances were also implemented in Sudan and Uganda; currently, both are the only African countries where ivermectin MDA was safely stopped in several transmission zones.

**Conclusions:**

Programmatic treatment and evaluation approaches, pioneered in the Americas, are the most efficient among the existing tools for elimination, and their broader use could catalyze the successful elimination of this disease in Africa.

**Electronic supplementary material:**

The online version of this article (10.1186/s40249-019-0557-1) contains supplementary material, which is available to authorized users.

## Multilingual abstracts

Please see Additional file 1 for translations of the abstract into the six official working languages of the United Nations.

## Background

The recent publication “Is onchocerciasis elimination in Africa feasible by 2025: a perspective based on lessons learnt from the African control programmes” by Dadzie et al. [1] is quite informative in recounting the evolution of onchocerciasis control programs in Africa and the history behind the decision to move from control to elimination. As pointed out by Dadzie et al. [1], onchocerciasis has long been the focus of the international community, due to its associated morbidities, including blindness, skin disease and an association with childhood epilepsy [2]. However, in several instances, Dadzie et al. mischaracterizes the contributions that the regional initiative to eliminate onchocerciasis in the Americas (OEPA) has made to the broader effort in Africa. We believe that key findings of OEPA’s experience have either been overlooked by the onchocerciasis control and elimination programs in Africa or the nuances of the situation in Latin America were not completely understood by policy makers. Further, Dadzie et al. express concern that Africa has been unduly “influenced by the relatively limited American experience.” Here, we focus on several key examples raised in that article and offer comment based on decades of OEPA’s experience and examples from the published, refereed literature, which we feel could inform and guide the African programs. A serious effort to eliminate onchocerciasis was started in by OEPA 1993. 25 years later, four American countries have been verified by World Health Organization (WHO) to have successfully interrupted the transmission of *Onchocerca volvulus*, the causative agent of onchocerciasis. Thus, we believe there are important contributions from the American experience that could benefit many African countries. Indeed, the American experience with onchocerciasis has played a leading role in the study of this disease throughout history. The connection between onchocerciasis and ocular disease was first discovered in the Americas, by the Guatemalan investigator Dr. Rodolfo Robles in 1919 [3], followed later by the link between onchocerciasis and epilepsy reported by Dr. Casis-Sacre in Mexico in 1938 [4]. These seminal clinical findings re-enforce the region’s historical, long-standing focus on this important disease of poverty.

## Main text

### The use of twice per year mass drug administration (MDA) of ivermectin

OEPA pioneered the use of twice per year treatments at high coverage rates, and in so doing has eliminated onchocerciasis from four of the six countries affected in the region; Colombia [5], Ecuador [6], Mexico [7] and Guatemala [8]. This scale up strategy in the American region from once to twice per year MDA began around 2000, based on a series of earlier studies first in Africa [9–12] and a 3 year pilot study in Guatemala [13] confirming the effectiveness of this regimen. These African and American studies formed the basis for the OEPA strategy of transmission elimination using twice per year ivermectin treatment. Consequently, more than 500 000 people no longer need ivermectin in the Americas [14] and children born in those 11 formerly endemic foci over the past decade are free of the risk of onchocerciasis and its associated pathologies. This effort represents a significant contribution towards the prevention of blindness globally. Using similar strategies and metrics, even greater numbers of individuals from Sudan and Uganda have been freed from the risk of onchocerciasis-related skin and eye disease as well.

It should be noted that the Onchocerciasis Control Program of West Africa (OCP) was also using a strategy of administration of ivermectin twice per year in many areas at the same time OEPA was doing so [15]. While OCP’s core activity was vector control, it had the same end goal as OEPA - interrupting parasite transmission. The main difference between these programs and the African Programme for Onchocerciasis Control (APOC) was that OEPA consistently chose to deploy twice per year ivermectin treatments in all endemic communities. In contrast, APOC sought to achieve sustained morbidity control by targeting only communities with the highest infection rates (> 20% nodule prevalence in adult males). The APOC strategy to control onchocerciasis as a public health problem was predicated on community directed annual treatments. APOC continued to advocate this annual treatment strategy even after the goal shifted to elimination of transmission rather than control. Thus, as reported in the final external review of the program [16], APOC was not successful in making a transition from a morbidity control program to a transmission elimination program. Outside of research and settings where vector control has historically been implemented by OCP, APOC never stopped treatments in an area where annual treatment was the primary intervention employed.

In December 31, 2015, APOC published a report describing strategic options and alternative treatment strategies for accelerating the elimination of onchocerciasis in Africa, and proposed the use of twice per year MDA, a core strategy developed and implemented by OEPA [17]. As noted above, two African countries (Sudan and Uganda) had already embraced the OEPA strategy [18–20] and demonstrated the elimination of transmission of *O. volvulus* in at least two endemic zones. Indeed, we contend that the twice per year MDA concept and the successes from the Americas became a foundational experience that moved those African programs from a control to an elimination paradigm.

### Stop MDA assessments based on serological testing rather than reliance on skin snip microscopy

Another long-standing OEPA strategy included serological testing and exclusion of skin snip microscopy in its assessments to determine the safe stoppage of MDA. This approach was incorporated into the recent WHO Guidelines for Stopping Mass Drug Administration and Verifying Elimination of Human Onchocerciasis: Criteria and Procedures (Guidelines) [21], which were developed by the Guidelines Development Group, composed of experts in onchocerciasis. To ensure intellectual rigor, these recommendations were also vetted through a systematic review process conducted by independent Grading of Recommendations Assessment, Development and Evaluation (GRADE) methodologists at WHO. Therefore, the recommendations in the 2016 Guidelines were systematically reviewed at two levels. Both skin snip microscopy and serology were determined to have “low certainty of evidence” in this process; however, the serological assay was given a “strong recommendation” while skin snip microscopy was given a “conditional recommendation”. Since the publication of the 2016 Guidelines, there have been additional reports comparing the performance of skin snip microscopy to polymerase chain reaction (PCR) amplification of parasite DNA from skin snips [22, 23], showing that skin snip microscopy significantly under-detects infections, with more pronounced deficits when *O. volvulus* microfilariae loads were low, which is the situation that programs encounter in hypo-endemic areas and in areas under successful MDA. These recent peer-reviewed reports reinforce the 2016 Guidelines recommendation that skin snip microscopy is not adequate to demonstrate the interruption of transmission of onchocerciasis. The current OV-16 serological assay is not perfect; it has to be used within very specific age range and employ statistically meaningful sample sizes to be informative. However, when used as recommended, it reflects the cumulative incidence of infection in the sampled population, and in conjunction with entomology helps determine whether transmission of *O. volvulus* has been interrupted. For the latter, the prevalence of black flies carrying infective larvae (L_3_s) in the head of less than 0.1% in parous flies or less than 0.05% in all flies (assuming a parity rate of 50%) are criteria used for entomological assessments. While the entomological metrics are difficult and expensive to achieve using conventional techniques, molecular methods (e.g. pool screen PCR [24]) and recently reported community directed methods for collecting vectors that can replace human landing collections [25] have made achieving the metrics set out in the guidelines both practical and affordable.

### Ivermectin as an *O. volvulus* macrofilaricide

Dr. Brian Duke and others working in the Americas pioneered the concept that repeated ivermectin treatments (twice or 4 times a year) were macrofilaricidal and that the impact of ivermectin on adult *O. volvulus* was best observed under conditions in which parasite transmission had been interrupted [26, 27]. Further, the long-term operational effect of repetitive twice/year treatments on adult worm survival and mating in the Americas showed that semi-annual treatments over 6–7 years at high coverage rates were generally equivalent to 10–13 years of vector control. This information was reported in 2004 [28]. Unfortunately, these seminal American research publications did not receive the attention they perhaps deserved by the African programs, leading some to unfairly conclude that the American experience was “relatively limited.”

### Epidemiological models

Dadzie et al. highlight the fact that APOC relied heavily on models to inform their strategy. In fact, both OCP and APOC worked closely with modelers to develop predictions for the effects of vector control and ivermectin MDA on the dynamics of infection in the human population and transmission of *O. volvulus*. This collaboration resulted in the development of at least three different models for onchocerciasis predictions: The Computer Simulation Program for Transmission and Control of Onchocerciasis (ONCHOSIM), EpiOncho and the Simulation Model of Onchocerciasis (SIMON) [29, 30]. For the Americas, OEPA adapted one of these models (SIMON) for use in Latin America as early as 2003, designating this modified model SIMONA, or Simulation Model of Onchocerciasis in the Americas [31]. Thus, the American and African programs both attempted to develop and utilize models to inform their decisions. Of course, the value of relying on models to make such strategic decisions is predicated on the underlying accuracy of the model predictions. In this regard, it is disturbing that the two models developed with APOC and OCP support (EPIONCHO and ONCHOSIM) have produced predictions that varied widely in some operationally important areas, such as the risk of recrudescence of infection following cessation of annual ivermectin distribution in areas where vector biting rates are high [32]. Additional work is needed to determine why this is the case, and to attempt to harmonize the results obtained by the models. Until this is done, it is difficult to determine which of these African prediction models should be used to inform important programmatic decisions, such as when it is safe to discontinue treatments. Furthermore, in the only operational test of the validity of the predictions of the models and epidemiological criteria relied upon by APOC, transmission was found to be ongoing in 3/5 countries where APOC’s criteria had indicated elimination had been achieved [33]. These findings suggest that the methods and metrics developed by APOC may inaccurately predict programmatic success at a high frequency.

### Elimination operational algorithms

OEPA’s original objective was to reach a point when ivermectin treatment could be successfully and safely withdrawn. Given the encouraging progress in three of the six endemic countries at the time, the annual InterAmerican Conference on Onchocerciasis (IACO) in 1996 concluded that the development of internationally accepted standards for certification of onchocerciasis transmission elimination were urgently needed [34]. [NB: WHO originally used the term “certification” to indicate institutional approval but has since chosen to use “verification” in its place.] OEPA’s steering committee (the Program Coordinating Committee, PCC) began to draft these criteria in 1997. This work included the conceptual control/elimination algorithm recently cited in Dadzie et al. [1]. During the same period, President Jimmy Carter pressed then WHO Director General Gro Harlem Brundtland for leadership on this issue. WHO headquarters rejected the idea of having elimination guidelines that were only applicable to the Americas Region, and convened a consultative meeting in Geneva in 2000 to develop global guidelines that involved experts from OCP, OEPA, and APOC. The baseline document for this meeting was written by OEPA PCC members Ed Cupp, Richard Collins, and Frank Richards. The OEPA based algorithms later appeared in the first WHO Guidelines for Certification of Elimination of Human Onchocerciasis: Criteria and Procedures [35]. Dadzie et al. [1] chose to adopt this algorithm for both the OCP (# 1) and APOC (# 3) figures, endorsing, perhaps unknowingly, OEPA’s seminal role in this process. APOC rarely if ever cited the 2001 WHO onchocerciasis elimination document, despite the fact that it was officially endorsed by WHO as a global strategy and published by WHO in English, French, Portuguese and Spanish.

### Vectorial capacity

When compared to much of Africa, onchocerciasis in the Americas was generally of lower intensity. However, many American foci were hyperendemic for the disease because of the extremely high annual biting rates by less efficient vector black flies due to their cibarial armature (*Simulium ochraceum* in Guatemala and Mexico, for example). However, *S. exiguum* in Ecuador has an equivalent vectorial capacity to savannah *S. damnosum* species [36], and hyperendemic communities in Ecuador had baseline community prevalences of > 90%, a situation very similar to that seen in the most hyper-endemic African settings. Yet *O. volvulus* transmission was eliminated in Ecuador after 9 years of twice per year treatment [6]. Around the same time as MDA was halted in Ecuador, the 2009 Diawara study in Mali and Senegal proclaimed the proof of principle of elimination of onchocerciasis in Africa with ivermectin mass treatment alone [15]. Unfortunately, Diawara et al. were unable to determine whether twice per year treatment could accelerate the process to under 15 years [15]. The Ecuador results would have helped shed light on this, but the relevancy of the success in Ecuador was not fully appreciated by the APOC community. Indeed, the Mali/Senegal report (which soon gave way to APOC elimination guidelines) also made no mention of the official 2001 WHO published guidelines, and especially ignored the principle of monitoring infection in children as a measure of recent incidence.

## Conclusions

OEPA’s experience, and its final success (or failure) will continue to provide lessons of the end game that should be appreciated by current African programs as their quest for transmission elimination continues. The American programs are now challenged with eliminating transmission in all areas, and strengthening both post-treatment and post-elimination surveillance. For example, OEPA is now engaged in the use of doxycycline and 4 times per year ivermectin treatment as new tools to assure finality of elimination. Some African programs will soon reach the point where OEPA is now, having stopped 94% of its treatments. Thus, African program managers ought to look carefully at the OEPA experience to discover and learn from past successes and potentially pertinent new approaches to dealing with non-technical issues of population migration, political instability, insecurity, cross border challenges, and waning political and financial support that are so fundamental to address in order to finish the job. We urge them to do so.

## Additional file

Additional file 1 Multilingual abstracts in the six official working languages of the United Nations. (PDF 291 kb)

## Data Availability

Not applicable.

## Abbreviations

APOC: African Programme for Onchocerciasis Control
GRADE: Grading of Recommendations Assessment, Development and Evaluation
IACO: Inter-American Conference on Onchocerciasis
MDA: Mass drug administration of ivermectin
OCP: Onchocerciasis Control Programme
OEPA: Onchocerciasis Elimination Program for the Americas
PCC: Program Coordinating Committee
WHO: World Health Organization
