# Supplementary material for: Elimination of onchocerciasis in Africa by 2025: the need for a broad perspective
Source: Infect Dis Poverty. 2019 Jul 15;8:50. doi: 10.1186/s40249-019-0557-1 (PMC6628485; doi:10.1186/s40249-019-0557-1)
Supplement: Supplementary file 1 — Multilingual abstracts in the six official working languages of the United Nations. (PDF 291 kb) [file 40249_2019_557_MOESM1_ESM.pdf]

Translation of the abstract into the five official working languages of the United Nations

واسع منظور إلى الحاجة :2025ب افريقيا في المرض كلابية داء علي القضاء

Ed Cupp, Mauricio Sauerbrey, Vitaliano Cama, Mark Eberhard, Patrick J. Lammie, Thomas R. Unnasch

مجرده
عام بحلول تحقيقه يمكن افريقيا في المرض كلابية داء علي القضاء" المعنون الأخير المنشور علي ردا :الخلفية المهم من ، وآخرون دادزي قبل من "الافريقيه المراقبة برامج من المستفادة الدروس إلى يستند منظور :2025 داء علي العالمي القضاء نحو الامريكيه التجربة من فيها لبس لا تنفيذيه ومساهمات بحوث الايجابيه وإبراز توضيح (الأنهار عمي) الإنسان كلابية.

الرئيسي النص:
عن فضلا ، السنه في ايفرمكتين من للادويه الشاملة الاداره جولات من أكثر أو مرتين استراتيجيات سمحت وقد انتقال علي للقضاء العالمية الصحة منظمه قبل من منها التحقق ليتم امريكيه بلدان أربعه OV-16 المصليات استخدام ، حاليا ؛ والسودان أوغندا في أيضا التطورات هذه ونفذت .للمرض المسبب الوكيل ، اونشوكيركا من الفرج .الإرسال مناطق من العديد في بأمان MDA ايفيرمكتين إيقاف تم حيث الوحيدة الافريقيه البلدان هي وكلاهما
القائمة الادات أكفا هي ، الأمريكتين في رائده كانت التي ، والتقييم للمعالجة البرنامجية النهوج ان :الاستنتاجات .افريقيا في المرض هذا علي الناجح القضاء يحفز ان يمكن أوسع نطاق علي واستخدامها ، منها للتخلص

Translated from English version into Arabic by Hamza Faleh Hamza, through

非洲2025年消除盘尾丝虫病：全局视角

Ed Cupp, Mauricio Sauerbrey, Vitaliano Cama, Mark Eberhard, Patrick J. Lammie, Thomas R. Unnasch

摘要
引言：为回应Dadzie等人发表的“非洲2025年消除盘尾丝虫病的可行性：基于非洲控制项目的经验教训”，本文旨在阐明和强调美国经验在全球消除人盘尾丝虫病（河盲症）方面的积极作用和贡献。
正文：每年使用两次或多次的伊维菌素群体化疗方案（MDA）以及基于OV-16的血清学方法，使四个美洲国家消除旋盘尾丝虫并得到世界卫生组织认可。目前苏丹和乌干达也在实施这些干预措施，这是在几个流行区中仅有的两个安全停止使用伊维菌素的非洲国家。
结论:在现有的盘尾丝虫病消除策略中，美洲率先采用的治疗方案和评估方法是最有效的，广泛实施这些措施可促进在非洲成功消除这一疾病。

Translated from English version into Chinese by Peng Song, edited by Jin Chen

Élimination de l'onchocercose en Afrique d'ici 2025: nécessité d'une perspective large.

Ed Cupp, Mauricio Sauerbrey, Vitaliano Cama, Mark Eberhard, Patrick J. Lammie, Thomas R. Unnasch

Résumé

Contexte : En réponse à la récente publication «L'élimination de l'onchocercose en Afrique est-elle réalisable d'ici 2025: une perspective fondée sur les enseignements tirés des programmes de lutte en Afrique » par Dadzie et al., contributions de l'expérience américaine à l'élimination mondiale de l'onchocercose humaine (cécité des rivières).

Texte principal : Les stratégies de deux ou plusieurs séries d'administration massive d'ivermectine par an, ainsi que l'utilisation de la sérologie OV-16, ont permis à l'Organisation mondiale de la santé de vérifier que quatre pays américains avaient éliminé la transmission *d'Onchocerca volvulus,* l'agent étiologique. Ces avancées ont également été mises en œuvre au Soudan et en Ouganda; actuellement, ils sont tous deux les seuls pays africains où l'ivermectine MDA a été arrêtée en toute sécurité dans plusieurs zones de transmission.

Conclusions : Les approches de traitement et d'évaluation programmatiques, mises au point dans les Amériques, sont les plus efficaces parmi les outils d'élimination existants, et leur utilisation plus large pourrait catalyser l'élimination réussie de cette maladie en Afrique.

Translated from English version into French by Laurine Crevoisier, Revised by Ahmad Dabaghzadeh, through

TRANSLATORS WITHOUT BORDERS

Ликвидация случаев онхоцеркоза в Африке к 2025 г.: необходимость широкого подхода

Эд Капп, Маурицио Сауэрбрей, Виталиано Кама, Марк Эберхард, Патрик Дж. Лэмми, Томас Р. Уннаш

Реферат

Предпосылки: В продолжение недавней публикации "Реализуема ли ликвидация случаев онхоцеркоза в Африке к 2025 г.: перспектива с точки зрения опыта, полученного в африканских программах профилактике" автора Дэдзи и др. важно пояснить и подчеркнуть вклад положительного и безоговорочного исследования и оперативных результатов американского опыта во всемирное устранение случаев онхоцеркоза у человека («речная слепота»).

Основной текст. Стратегии двух или более циклов массового введения препарата (МВП) ивермектина в год, а также использование серологических тестов с OV-16 позволило четырем американским странам получить верификацию Всемирной организации здравоохранения по ликвидации передачи возбудителя *Onchocerca volvulus*, являющегося этиологическим агентом. Эти наработки были также внедрены в Судане и Уганде. На

данный момент это единственные африканские страны, где в нескольких зонах распространения болезни было успешно прекращено МВП ивермектина.

Выводы: Подход на основе программного лечения и оценки, который начали внедрять в Южной и Северной Америках, являются наиболее эффективными среди существующих инструментов устранения, а его широкое применение может ускорить успешную ликвидацию этой болезни в Африке.

Translated from English version into Russian by Veronika Demeshchik, Revised by Alexander Somin, through

La eliminación de la oncocercosis en África antes de 2025: la necesidad de una perspectiva amplia.

Ed Cupp, Mauricio Sauerbrey, Vitaliano Cama, Mark Eberhard, Patrick J. Lammie, Thomas R. Unnasch

Resumen

Antecedentes: en respuesta a la reciente publicación «Is onchocerciasis elimination in Africa feasible by 2025: a perspective based on lessons learnt from the African control programmes» (¿Es factible la eliminación de la oncocercosis en África antes de 2025?: una perspectiva basada en las lecciones aprendidas de los programas de control africanos) de Dadzie et al., resulta importante aclarar y destacar la investigación positiva e inequívoca y las contribuciones operativas de la experiencia en América con miras a la eliminación a nivel mundial de la oncocercosis humana (ceguera de los ríos).

Texto principal: las estrategias de dos o más rondas de administración masiva de medicamentos (MDA, por sus siglas en inglés) de ivermectina al año, así como el uso de serología OV-16 han permitido que la Organización Mundial de la Salud verificase que cuatro países americanos han eliminado la transmisión de *Onchocerca volvulus*, el agente etiológico. Estos avances se han implementado asimismo en Sudán y en Uganda; actualmente, estos son los únicos países africanos en los que se suspendió de manera segura la MDA de ivermectina en varias zonas de transmisión.

Conclusiones: los enfoques programáticos de tratamiento y evaluación, iniciados en las América, son los más eficientes de entre las herramientas existentes para la eliminación, y su uso más amplio podría catalizar la exitosa eliminación de esta enfermedad en África.

Translated from English version into Spanish by Mayra León, Revised by Gabriela Allant, hrough
